# Cytological Studies of 25 Species and Four Varieties of *Artemisia* (Asteraceae) from China, Toward a Better Understanding of the Variation Patterns of Chromosomes in the Genus

**DOI:** 10.3390/plants14081253

**Published:** 2025-04-20

**Authors:** Xinqiang Guo, Yiran Jiang, Xianxiang Zeng, Fuhui Tan, Dawei Xue, Yuhuan Wu

**Affiliations:** 1 College of Life and Environmental Sciences, Hangzhou Normal University, Hangzhou 311121, China; xqguo@hznu.edu.cn (X.G.); 2022210301026@stu.hznu.edu.cn (Y.J.); dwxue@hznu.edu.cn (D.X.); 2 Zhejiang Provincial Key Laboratory for Genetic Improvement and Quality Control of Medicinal Plants, Hangzhou Normal University, Hangzhou 311121, China; 3 Liping County Bureau of Forestry, Liping 557300, China; 18386755587@163.com (X.Z.); lplytfh@163.com (F.T.)

**Keywords:** Asteraceae, chromosome number, cytology, karyotype, Qinghai–Tibetan Plateau

## Abstract

The chromosome numbers of 56 populations belonging to 25 species and 4 varieties of *Artemisia* L. (Asteraceae) from China were examined, and those of 13 species and four varieties are reported here for the first time. The karyotypes of 39 populations in 23 species and four varieties were also studied. Among them, twelve species and one variety were found to be diploid, with 2*n* = 16 or 18; nine species and three varieties were found to be tetraploid, with 2*n* = 32 or 36; and two species were found to have both diploid and tetraploid cytotypes. Two species were found to have aneuploid cytotypes. The karyotypes of *Artemisia* are similar, with most chromosomes belonging to median-centromeric (m) and a few belonging to submedian-centromeric (sm) or subterminal-centromeric (st). The high level of polyploids in *Artemisia* from the Qinghai–Tibetan Plateau indicates that polyploidy has played an important role in the evolutionary speciation of this highly diversified genus in this region.

## 1. Introduction

*Artemisia* L. (Asteraceae), a large genus of nearly 500 species, is mainly distributed in temperate regions of the Northern Hemisphere [1,2]. China, where more than 200 species have been described, is undoubtedly one of the most important distribution and diversity centers of the genus [3]. Species of *Artemisia* usually possess significant economic value. Among them, the most famous is *A. annua*, from which the anti-malarial artemisinin can be extracted [4,5]. In addition, *A. argyi* is a well-known traditional Chinese medicine reported to be effective in relieving swelling and pain [6].

Traditionally, this genus is divided into four generally accepted subgenera, i.e., subg. *Absinthium* (Miller) Less., subg. *Artemisia*, subg. *Dracunculus* (Besser) Rydb., and subg. *Seriphidium* Besser ex Less., on the basis of morphological characters, including the shape of the leaflets and capitula and the fertility of the florets [3,7]. However, recent molecular phylogenetic studies have revealed that the infra-generic divisions of *Artemisia* are not natural. All the subgenera are polyphyletic [7,8]. The species delimitations within *Artemisia* are also rather problematic [9,10,11,12]. This suggests that the phylogenetic relationships and evolutionary history of *Artemisia* are complex. In order to produce a more reasonable taxonomic treatment and phylogeny for the genus, a thorough survey of different kinds of characters is needed.

Chromosomes play an important role in plant taxonomy and systematics. Before sound taxonomic and systematic decisions can be made, the variation pattern of chromosome number and karyotype should be understood fully [13,14]. Cytological studies on *Artemisia* have been conducted in various countries, revealing that chromosome data varies significantly within the genus. Two basic chromosome numbers are generally accepted in *Artemisia*, *x* = 8 and 9, with *x* = 9 being the most common [15,16,17]. Different chromosome numbers and a high rate of polyploidy have also been consecutively reported in *Artemisia*, with 2*n* = 16, 18, 32, 34, 36, 72, 90, 108, and 144. The diploid 2*n* = 2*x* = 18 and the tetraploid 2*n* = 4*x* = 36 are the most frequent [15,16]. Some species with wide distributions even have populations with different chromosome numbers and polyploidy levels. For example, *A. dracunculus* includes populations with 2*n* = 2*x* = 18, 2*n* = 4*x* = 36, 2*n* = 6*x* = 54, and 2*n* = 10*x* = 90 [18,19,20,21,22,23]. Populations of *A. japonica* Thunb. from Japan, South Korea, and Russia have been found to be tetraploid (2*n* = 4*x* = 36) [24,25,26,27,28], while plant individuals from India and Yunnan in China are diploid (2*n* = 2*x* = 18) [29,30]. Additionally, aneuploidy is frequently reported in some species. For example, *A. verlotiorum* has primarily two cytotypes, 2*n* = 48 and 54 [31,32,33], but aneuploidy, in which chromosome numbers are 2*n* = 50 and 52, has also been documented [32].

The Qinghai–Tibetan Plateau (QTP) harbors nearly 12,000 species of vascular plants; it has high mountains and extreme environments and possesses exceptional species richness and high levels of endemism [34,35,36,37]. Polyploidy is considered one of the most important mechanisms in plant evolution [13,38]. It is relatively common in plants growing in alpine and arctic regions with harsh environments and cold climates [39,40]. Thus, a high rate of polyploidy can be expected in the QTP [34,41,42]. Yuan and Yang [43] and Chen et al. [44] found that polyploidy has played an important role in the speciation of *Aconitum* subg. *Lycoctonum* (DC.) Peterm. (Ranunculaceae) and *Buddleja* L. (Loganiaceae), respectively. However, in the QTP endemic genera *Cremanthodium* Benth. (Asteraceae) [45], *Delphinium* L. (Ranunculaceae) [42], and *Ligularia* Cass. (Asteraceae) [46], low proportions of polyploidy have been found. Considering the high species diversity and the small proportion of species with available chromosomal data, the role played by polyploidy in the speciation of this region is still unclear. More plant groups with possibly different evolutionary histories should be cytologically studied to gain a better understanding of the speciation patterns in this region.

To date, the *Artemisia* species from China are still not cytologically well known, with only c. 14 species, mainly from northeastern China, having detailed chromosomal data [28,30]. Species from the QTP, a diversity center of *Artemisia*, have rarely been studied, especially those endemic to this region. Given the wide distribution of *Artemisia* and the frequent chromosomal variation among populations, it is necessary to accumulate further cytological data for the genus.

To comprehensively assess the chromosome variation pattern in *Artemisia* from China and evaluate the values in taxonomic and phylogenetic studies, we conducted extensive samplings, particularly from the QTP in southwestern China, and analyzed their chromosome numbers and karyotypes.

## 2. Materials and Methods

All studied plants were collected during our field trips in Guangdong, Hebei, Henan, Hubei, Hunan, Qinghai, Shaanxi, Sichuan, Xinjiang, Xizang, and Zhejiang provinces in China (Table 1 and Table S1). These plants were cultivated in a nursery in the South China Botanical Garden, Chinese Academy of Sciences, until their roots could be harvested for cytological study. Voucher specimens were deposited in the Herbarium of South China Botanical Garden, Chinese Academy of Sciences (IBSC). The actively growing root tips were cut and pretreated in 1/100 colchicine solution at about 20 °C for 2.5 h in the dark and then fixed in Carnoy I (glacial acetic acid:absolute ethanol, 1:3) at 4 °C for 1 h. They were then macerated in a 1:1 mixture of 1 M hydrochloric acid (HCl) and 45% acetic acid at 37 °C for 40 min, stained in 1% aceto-orcein overnight, and squashed for chromosome observations. A Leica DM1000 microscope (Leica Microsystems, Wetzlar, Germany) was used to examine chromosomes and take photos.

Karyotype analysis was performed by measuring mitotic-metaphase chromosomes. The symbols used to describe the karyotypes followed Levan et al. [47]: m, median-centromeric chromosome with an arm ratio of 1.0–1.7; sm, submedian-centromeric chromosome with an arm ratio of 1.7–3.0; st, subterminal-centromeric chromosome with an arm ratio of 3.0–7.0; sat, satellite chromosomes. Karyotypes were classified according to Stebbins’ criteria [13].

Karyotype asymmetry was analyzed using the method proposed by Romero-Zarco [48]. The A1 value reflects intra-chromosomal karyotype asymmetry, where smaller A1 values indicate less difference between chromosome arms and greater symmetry in chromosomal internal structure. The A2 value represents inter-chromosomal karyotype asymmetry, with smaller A2 values signifying reduced length variation among chromosomes and lower karyotypic dimorphism.

## 3. Results

**Figure 1:**
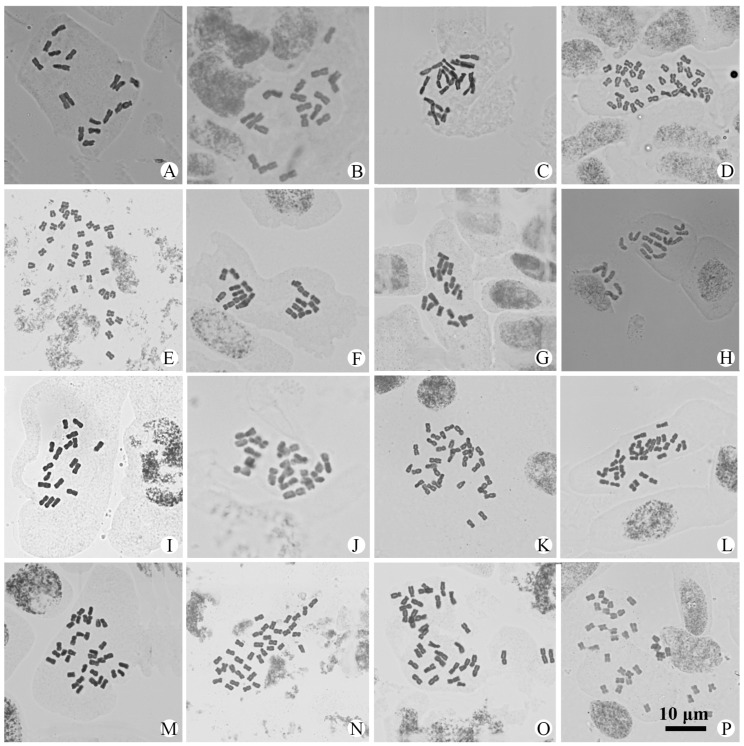
Photomicrographs of mitotic metaphase chromosomes in 16 populations representing 9 species and 1 variety of *Artemisia* from China. (**A**). *A. anomala*, 2*n* = 18; (**B**). *A. anomala*, 2*n* = 18; (**C**). *A. anomala*, 2*n* = 18; (**D**). *A. baimaensis*, 2*n* = 36; (**E**). *A. campbellii*, 2*n* = 36; (**F**). *A. divaricata*, 2*n* = 18; (**G**). *A. divaricata*, 2*n* = 18; (**H**). *A. divaricata*, 2*n* = 18; (**I**). *A. fulgens* var. *meiguensis*, 2*n* = 18; (**J**). *A. gmelinii*, 2*n* = 18; (**K**). *A. incisa*, 2*n* = 36; (**L**). *A. igniaria*, 2*n* = 34; (**M**). *A. imponens*, 2*n* = 36; (**N**). *A. imponens*, 2*n* = 36; (**O**). *A. imponens*, 2*n* = 36; (**P**). *A. jilongensis*, 2*n* = 36. All same scale.

**Figure 2:**
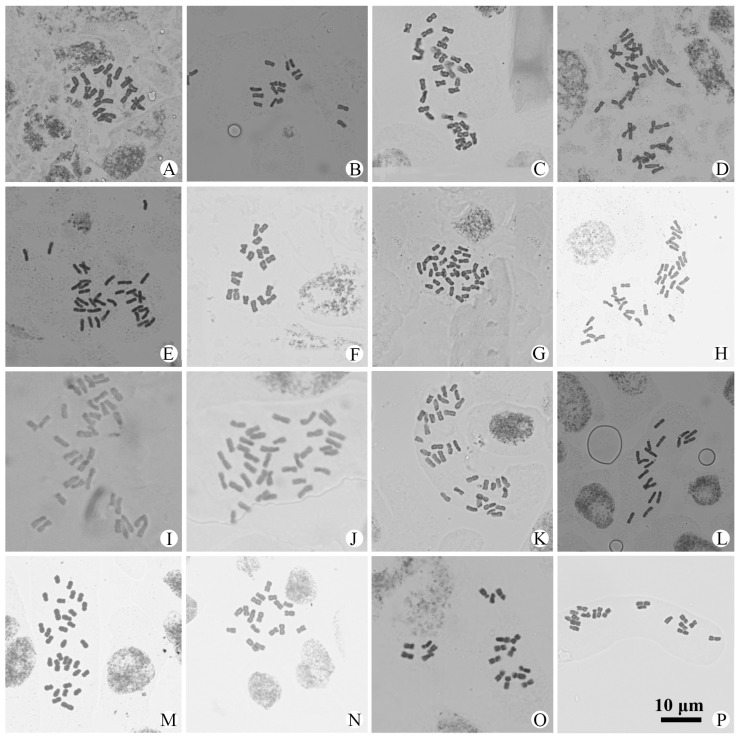
Photomicrographs of mitotic metaphase chromosomes in 16 populations representing 6 species and 1 variety of *Artemisia* from China. (**A**). *A. lactiflora*, 2*n* = 18; (**B**). *A. lactiflora*, 2*n* = 18; (**C**). *A. lactiflora*, 2*n* = 36; (**D**). *A. lactiflora* var. *incisa*, 2*n* = 36; (**E**). *A. lactiflora* var. *incisa*, 2*n* = 36; (**F**). *A. minor*, 2*n* = 18; (**G**). *A. moorcroftiana*, 2*n* = 36; (**H**). *A. moorcroftiana*, 2*n* = 36; (**I**). *A. moorcroftiana*, 2*n* = 36; (**J**). *A. moorcroftiana*, 2*n* = 36; (**K**). *A. phaeolepis*, 2*n* = 36; (**L**). *A. phaeolepis*, 2*n* = 18; (**M**). *A. princeps*, 2*n* = 32; (**N**). *A. qinlingensis*, 2*n* = 18; (**O**). *A. qinlingensis*, 2*n* = 18; (**P**). *A. qinlingensis*, 2*n* = 18. All same scale.

**Figure 3:**
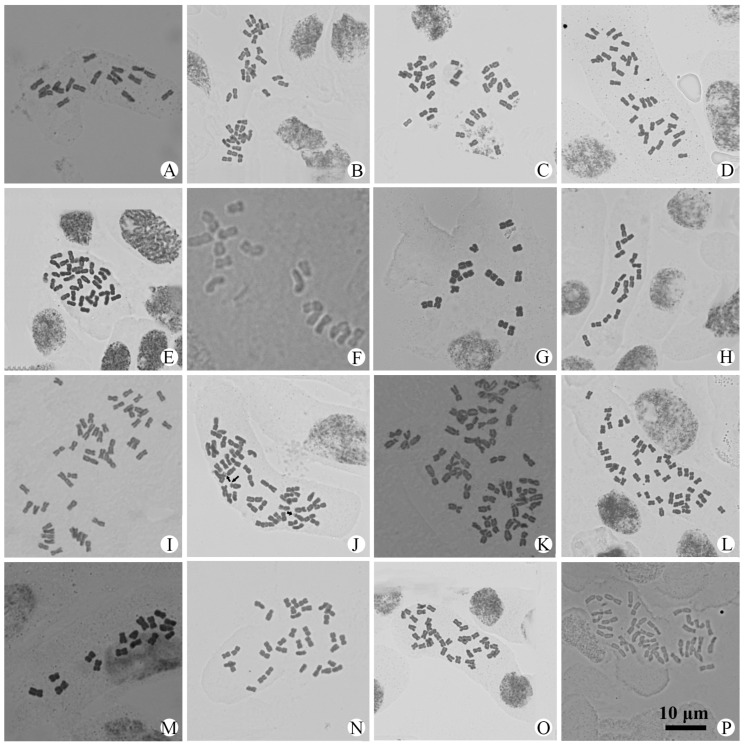
Photomicrographs of mitotic metaphase chromosomes in 16 populations representing 5 species and 2 varieties of *Artemisia* from China. (**A**). *A. sacrorum*, 2*n* = 18; (**B**). *A. tainingensis*, 2*n* = 36; (**C**). *A. tainingensis*, 2*n* = 36; (**D**). *A. tainingensis*, 2*n* = 36; (**E**). *A. tainingensis*, 2*n* = 36; (**F**). *A. verbenacea*, 2*n* = 16; (**G**). *A. verbenacea*, 2*n* = 16; (**H**). *A. verbenacea*, 2*n* = 16; (**I**). *A. verlotiorum*, 2*n* = 50; (**J**). *A. verlotiorum*, 2*n* = 50; (**K**). *A. verlotiorum*, 2*n* = 50; (**L**). *A. verlotiorum*, 2*n* = 50; (**M**). *A. vulgaris*, 2*n* = 16; (**N**). *A. vulgaris* var. *xizangensis*, 2*n* = 36; (**O**). *A. waltonii* var. *yushuensis*, 2*n* = 36; (**P**). *A. waltonii* var. *yushuensis*, 2*n* = 36. All same scale.

**Figure 4:**
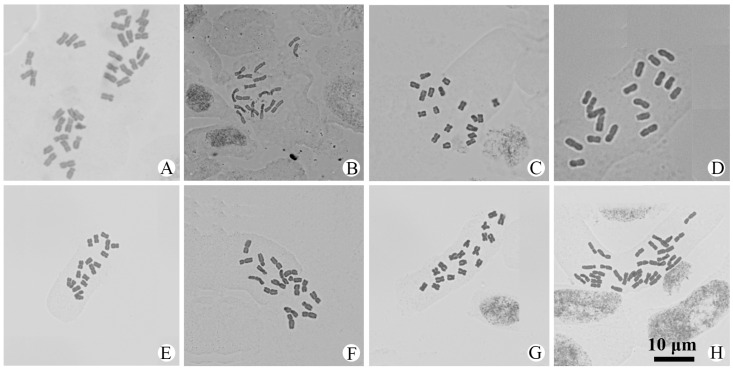
Photomicrographs of mitotic metaphase chromosomes in 16 populations representing 5 species and 1 variety of *Artemisia* from China. (**A**). *A. waltonii* var. *yushuensis*, 2*n* = 36; (**B**). *A. wellbyi*, 2*n* = 18; (**C**). *A. zayuensis*, 2*n* = 18; (**D**). *A. younghusbandii*, 2*n* = 18; (**E**). *A. youngii*, 2*n* = 18; (**F**). *A. youngii*, 2*n* = 18; (**G**). *A. youngii*, 2*n* = 18; (**H**). *A. yunnanensis*, 2*n* = 32. All same scale.

Chromosome numbers were obtained for 56 populations of 25 species and 4 varieties of *Artemisia* from China (Table 1 and Table S1). All of them are illustrated in Figure 1, Figure 2, Figure 3 and Figure 4. The chromosome numbers of 13 species and four varieties are reported here for the first time. Twelve species and one variety were found to be diploid, with 2*n* = 16 or 18; nine species and three varieties were found to be tetraploid, with 2*n* = 32 or 36; and two species were found to have both diploid and tetraploid cytotypes. Two species were found to be aneuploid. No small B chromosome was observed. We also analyzed the karyotypes of 39 populations in 23 species and 4 varieties. All the karyotypes are provided in Figures S1–S5 to illustrate the chromosome morphology and size. The karyotype formulae of the *Artemisia* studied with available karyotypic data are shown in Table 1. The karyotypes are somewhat uniform. Most of the chromosomes are median-centromeric (m), with a few submedian-centromeric (sm) or subterminal-centromeric (st).

### 3.1. Artemisia anomala *S. Moore*

This species is widely distributed in southern China. Three populations from Yingde in Guangdong, Xinning in Hunan, and Hangzhou in Zhejiang were examined. All three populations were found to be diploid, with a chromosome number of 2*n* = 18 (Figure 1A–C). The karyotypes of the populations from Yingde and Xinning were 2*n* = 16m + 2sm (Figure S1A) and 2*n* = 14m + 2sm + 2st (Figure S1B), and both belonged to Stebbins’ 2A type.

### 3.2. A. baimaensis *Y.R. Ling & Z.C. Chuo*

This species is endemic to Qinghai, China. One population from the type locality, Baima County, was examined and found to be tetraploid, with a chromosome number of 2*n* = 4*x* = 36 (Figure 1D). The karyotype consisted of 28 median-centromeric and 8 submedian-centromeric chromosomes, and it belonged to Stebbins’ 2A type. The karyotype was formulated as 2*n* = 28m + 8sm (Figure S1C). The chromosome number and karyotype of this species are reported here for the first time.

### 3.3. A. campbellii *Hook. f. & Thoms.*

This species is distributed in southern Xizang in China, Bhutan, and India. One population from Lhünzê County in Xizang was examined. It was found to be tetraploid, with a chromosome number of 2*n* = 4*x* = 36 (Figure 1E). The karyotype formula is 2*n* = 28m + 8st (Figure S1D). The chromosome number and karyotype of this species are reported here for the first time.

### 3.4. A. divaricata *(Pamp.) Pamp.*

This species is mainly distributed in western Sichuan, China. Three populations from Barkam, Jinchuan, and Zamtang counties were studied. All three populations were found to be diploid, with a chromosome number of 2*n* = 2*x* = 18 (Figure 1F–H). The karyotypes of two populations were uniform, consisting of 14 median-centromeric and 4 subterminal chromosomes (2*n* = 14m + 4st) (Figure S1E,F). The karyotypes all belonged to Stebbins’ 2A type. The chromosome number and karyotype of this species are reported here for the first time.

### 3.5. A. fulgens *var.* meiguensis *(Y.R. Ling) X.Q. Guo, L. Wang & Q.E. Yang*

This variety is endemic to Sichuan, China. One population from Ebian County was examined and found to be diploid, with 2*n* = 2*x* = 18 (Figure 1I). The karyotype consisted of 14 median-centromeric and 4 submedian-centromeric chromosomes, and it belonged to Stebbins’ 2A type. The karyotype was formulated as 2*n* = 14m + 4sm (Figure S1G). The chromosome number and karyotype of this variety are reported here for the first time.

### 3.6. A. gmelinii *Web. ex Stechm.*

This species is widely distributed in temperate regions of the Northern Hemisphere. One population from Maduo in Qinghai, China, was examined and found to be diploid, with 2*n* = 2*x* = 18 (Figure 1J).

### 3.7. A. incisa *Pamp.*

This species is distributed in southern Xizang, China, and in Bhutan, Kashmir, India, Nepal, and Pakistan. One population from Dinggyê, Xizang, was examined and found to be tetraploid, with 2*n* = 4*x* = 36 (Figure 1K). The karyotype was formulated as 2*n* = 28m + 4sm + 4st (Figure S1H) and belonged to Stebbins’ 2A type.

### 3.8. A. igniaria *Maxim.*

This species is mainly distributed in northern China. One population from Xingtai, Hebei, was examined and found to be aneuploid, with 2*n* = 34 (Figure 1L). The karyotype consisted of 30 median-centromeric and 4 submedian-centromeric chromosomes and belonged to Stebbins’ 2A type (Figure S1I). No satellite chromosome was observed. To ensure the accuracy of the results, a total of fifteen cells (five cells from each plant) were carefully examined.

### 3.9. A. imponens *Pamp.*

This species is distributed only in southern Qinghai, western Sichuan, and eastern Xizang. Three populations from Sichuan (Hongyuan, Songpan, and Xiangcheng counties) were examined. All three populations were tetraploid, with 2*n* = 4*x* = 36 (Figure 1M–O). The karyotypes were formulated as 2*n* = 32m + 4sm (Figure S2A), 2*n* = 28m + 8st (Figure S2B), and 2*n* = 28m + 8sm (Figure S2C), and all belonged to Stebbins’ 2A type. The chromosome number and karyotype of this species are reported here for the first time.

### 3.10. A. jilongensis *Y.R. Ling & Humphries*

This species is only distributed in Xizang, China. One population from the type locality, Gyirong, was examined. It was found to be tetraploid, with a chromosome number of 2*n* = 4*x* = 36 (Figure 1P). The chromosome number of *A. jilongensis* is reported here for the first time.

### 3.11. A. lactiflora *Wall. ex DC.*

This species is distributed in southern China, India, Indonesia, Laos, and Singapore. Three populations were examined, with two populations from Sichuan being diploid (2*n* = 2*x* = 18) (Figure 2A,B) and one population from Hubei being tetraploid (2*n* = 4*x* = 36) (Figure 2C). The two populations from Sichuan had uniform karyotypes, consisting of 14 median-centromeric and 4 submedian-centromeric chromosomes (Figure S2D,E), and they belonged to Stebbins’ 2A type.

### 3.12. A. lactiflora *var.* taibaishanensis *X.D. Cui*

This variety is mainly distributed in central China. One population from Hubei (Shennongjia) and one from Sichuan (Tianquan) were examined. Both were tetraploid, with a chromosome number of 2*n* = 4*x* = 36 (Figure 2D,E). The karyotypes were formulated as 2*n* = 28m + 8st (Figure S2F) and 2*n* = 32m + 4sm (Figure S3A) and belonged to Stebbins’ 2A type. This variety was cytologically studied here for the first time.

### 3.13. A. minor *Jacq. ex Bess.*

This species is distributed in Qinghai, southern Xizang, China, and northern India. One population from Qinghai (Madoi) was examined and found to be diploid, with 2*n* = 2*x* = 18 (Figure 2F). The karyotype was formulated as 2*n* = 14m + 4sm (Figure S3B) and belonged to Stebbins’ 2A type.

### 3.14. A. moorcroftiana *Wall. ex DC.*

This species is mainly distributed in the QTP. Four populations from Xizang (Baxoi, Bomi, Comai, and Zayü) were examined and found to be tetraploid, with 2*n* = 4*x* = 36 (Figure 2G–J). This represents a new cytotype for *A. moorcroftiana*. The karyotypes of populations from Baxoi and Bomi were formulated as 2*n* = 28m + 8sm (Figure S3C,D) and belonged to Stebbins’ 2A type.

### 3.15. A. phaeolepis *Krasch.*

This species is widely distributed in China. One population from Maqén, Qinghai, and one population from Huocheng, Xinjiang, were examined and found to be tetraploid, with 2*n* = 4*x* = 36 (Figure 2K), and diploid, with 2*n* = 2*x* = 18 (Figure 2L), respectively. The karyotype of the population from Maqén consisted of 28 median-centromeric and 8 submedian-centromeric chromosomes and belonged to Stebbins’ 2A type (2*n* = 28m + 8sm) (Figure S3E). The population from Huocheng consisted of 12 median-centromeric and 6 submedian-centromeric chromosomes (2*n* = 12m + 6sm) (Figure S3F).

### 3.16. A. princeps *Pamp.*

This species is widely distributed in China, Japan, and Russia. One population from Luding, Sichuan, was examined and found to be tetraploid, with 2*n* = 4*x* = 32, representing another chromosome base number in the genus, *x* = 8 (Figure 2M). The karyotype was formulated as 2*n* = 28m + 4st (Figure S3G) and belonged to Stebbins’ 2A type. This cytotype is newly reported here.

### 3.17. A. qinlingensis *Ling & Y.R. Ling*

This species is distributed in Gansu, Henan, Hubei, and Shaanxi. Three populations were examined, and all were found to be diploid, with 2*n* = 2*x* = 18 (Figure 2N–P). The karyotype of the Luanchuan population was formulated as 2*n* = 14m + 4st (Figure S3H), and that of the Shennongjia population was formulated as 2*n* = 12m + 6sm (Figure S4A). Both belonged to Stebbins’ 2A type. The chromosome number and karyotype of this species are reported here for the first time.

### 3.18. A. sacrorum *Ledeb.*

The species is widely distributed in temperate regions of the Northern Hemisphere. One population from Ürümqi, Xinjiang, was studied and found to be diploid. The chromosome number was 2*n* = 18 (Figure 3A), with a karyotype formula of 2*n* = 14m + 4sm (Figure S4B). The karyotype belonged to Stebbins’ 2A type.

### 3.19. A. tainingensis *Hand.-Mazz.*

This species is only distributed in the QTP. Four populations were examined and found to be tetraploid, with 2*n* = 4*x* = 36 (Figure 3B–E). The karyotypes of two populations (one from Madoi in Qinghai and another from Aba in Sichuan) were examined, with formulae of 2*n* = 28m + 4sm + 4st (Figure S4C) and 2*n* = 28m + 8st (Figure S4D). The karyotypes belonged to Stebbins’ 2A type. The chromosome number and karyotype of this species are reported here for the first time.

### 3.20. A. verbenacea *(Komar.) Kitag.*

This species is distributed in northern China. Three populations were examined (Table 1). All populations were found to be diploid, with 2*n* = 2*x* = 16 (Figure 3F–H). The karyotype formulae were 2*n* = 14m + 2sm for the Dawu population (Figure S4E) and 2*n* = 14m + 2st for the Jiuzhaigou and Qamdo populations (Figure S4F,G). All karyotypes belonged to Stebbins’ 2A type. The chromosome number and karyotype of this species are reported here for the first time.

### 3.21. A. verlotiorum *Lamotte*

This species is widely distributed in the Northern Hemisphere. Four populations were examined (Table 1 and Table S1). All four populations were found to be aneuploid, with chromosome numbers of 2*n* = 50 (Figure 3I–L). The karyotype of the Lushi population was formulated as 2*n* = 40m + 10st (Figure S4H) and belonged to Stebbins’ 2A type.

### 3.22. A. vulgaris *L.*

This species is widely distributed in the Northern Hemisphere. One population from Ürümqi, Xinjiang, was examined and found to be diploid, with 2*n* = 2*x* = 16 (Figure 3M). The karyotype was formulated as 2*n* = 14m + 2st (Figure S5A) and belonged to Stebbins’ 2A type. The chromosome number and karyotype of the population from China are reported here for the first time.

### 3.23. A. vulgaris *var.* xizangensis *Ling & Y.R. Ling*

This variety is endemic to Xizang, China. One population from Bomi County was examined and found to be tetraploid, with 2*n* = 4*x* = 36 (Figure 3N). The karyotype was formulated as 2*n* = 28m + 4sm + 4st (Figure S5B) and belonged to Stebbins’ 2A type. The chromosome number and karyotype of this variety are reported here for the first time.

### 3.24. A. waltonii *var.* yushuensis *Y.R. Ling*

This variety is distributed in southern Qinghai, western Sichuan, and eastern Xizang. Three populations were examined (Table 1 and Table S1). All of them were tetraploid, with a chromosome number of 2*n* = 4*x* = 36 (Figure 3O,P and Figure 4A). The karyotype of the Chindu population was formulated as 2*n* = 28m + 8sm (Figure S5C) and belonged to Stebbins’ 2A type. The chromosome number and karyotype of this variety are reported here for the first time.

### 3.25. A. wellbyi *Hemsl. & Pears. ex Deasy*

This species is distributed in southern Xizang, China, and India. One population from Dinggyê was examined and found to be tetraploid, with a chromosome number of 2*n* = 2*x* = 18 (Figure 4B). The karyotype was formulated as 2*n* = 14m + 2sm + 2st (Figure S5D) and belonged to Stebbins’ 2A type. The chromosome number and karyotype of this species are reported here for the first time.

### 3.26. A. zayuensis *Ling & Y.R. Ling*

This species is endemic to Xizang and Yunnan. One population from Lhünzê County in Xizang was examined, and it is diploid with a chromosome number of 2*n* = 18 (Figure 4C). The karyotype was formulated as 2*n* = 12m + 6sm (Figure S5E) and belonged to Stebbins’ 2A type. The chromosome number and karyotype of this species are reported here for the first time.

### 3.27. A. younghusbandii *J.R. Drumm. ex Pamp.*

This species is endemic to Xizang. One population from Kangmar was examined and found to be diploid, with a chromosome number of 2*n* = 2*x* = 18 (Figure 4D). The karyotype was formulated as 2*n* = 14m + 4sm (Figure S5F) and belonged to Stebbins’ 2A type. The chromosome number and karyotype of this species are reported here for the first time.

### 3.28. A. youngii *Y.R. Ling*

This species is only distributed in southern Qinghai and eastern Xizang. One population from Qinghai and two populations from Xizang were examined (Figure 4E–G). All were diploid, with chromosome numbers of 2*n* = 2*x* = 18. Analysis of the karyotype was conducted for the Baxoi population, and the karyotype was formulated as 2*n* = 12m + 6sm (Figure S5G) and belonged to Stebbins’ 2A type. The chromosome number and karyotype of this species are reported here for the first time.

### 3.29. A. yunnanensis *J.F. Jeffrey ex Diels*

This species is distributed in Sichuan and Yunnan, China. One population from Danba, Sichuan, was examined. This population is tetraploid, with 2*n* = 4*x* = 32 (Figure 4H). The karyotype formula is 2*n* = 24m + 4sm + 4st (Figure S5H), and it belonged to Stebbins’ 2B type. The chromosome number and karyotype of this species are reported here for the first time.

**Table 1:** Chromosome number, karyotype formulae, total chromosome length (TKL), intra-chromosomal asymmetry (A1), inter-chromosomal asymmetry (A2), and Stebbins’ chromosomal asymmetry (ST) in each taxon investigated within *Artemisia* from China.

Taxon	2*n*	Karyotype Formula	TKL (μm)	A_1_	A_2_	ST	Figure
*A. anomala* S. Moore	18	2*n* = 16m + 2sm	63.06	0.26	0.14	2A	1A, S1A
*A. anomala* S. Moore	18	2*n* = 14m + 2sm + 2st	60.27	0.32	0.15	2A	1B, S1B
*A. anomala* S. Moore	18						1C
*A. baimaensis* Y.R. Ling & Z.C. Chuo	36	2*n* = 28m + 8sm	110.74	0.30	0.13	2A	1D, S1C
*A. campbellii* Hook. f. & Thoms.	36	2*n* = 28m + 8st	102.81	0.33	0.09	2A	1E, S1D
*A. divaricata* (Pamp.) Pamp.	18	2*n* = 14m + 4st	61.50	0.35	0.12	2A	1F, S1E
*A. divaricata* (Pamp.) Pamp.	18	2*n* = 14m + 4st	61.70	0.29	0.12	2A	1G, S1F
*A. divaricata* (Pamp.) Pamp.	18						1H
*A. fulgens* var. *meiguensis* (Y.R. Ling) X.Q. Guo, L. Wang & Q.E. Yang	18	2*n* = 14m + 4sm	62.55	0.27	0.12	2A	1I, S1G
*A. gmelinii* Web. ex Stechm.	18						1J
*A. incisa* Pamp.	36	2*n* = 28m + 4sm + 4st	116.14	0.33	0.12	2A	1K, S1H
*A. igniaria* Maxim.	34	2*n* = 30m + 4sm	102.16	0.23	0.15	2A	1L, S1I
*A. imponens* Pamp.	36	2*n* = 32m + 4sm	118.05	0.27	0.11	2A	1M, S2A
*A. imponens* Pamp.	36	2*n* = 28m + 8st	112.46	0.34	0.09	2A	1N, S2B
*A. imponens* Pamp.	36	2*n* = 28m + 8sm	105.74	0.33	0.14	2A	1O, S2C
*A. jilongensis* Y. R. Ling & Humphries	36						1P
*A. lactiflora* Wall. ex DC.	18	2*n* = 14m + 4sm	67.56	0.29	0.16	2A	2A, S2D
*A. lactiflora* Wall. ex DC.	18	2*n* = 14m + 4sm	59.35	0.35	0.16	2A	2B, S2E
*A. lactiflora* Wall. ex DC.	36						2C
*A. lactiflora* var. *taibaishanensis* X.D. Cui	36	2*n* = 28m + 8st	97.57	0.32	0.14	2A	2D, S2F
*A. lactiflora* var. *taibaishanensis* X.D. Cui	36	2*n* = 32m + 4sm	107.94	0.24	0.11	2A	2E, S3A
*A. minor* Jacq. ex Bess.	18	2*n* = 14m + 4sm	63.72	0.26	0.11	2A	2F, S3B
*A. moorcroftiana* Wall. ex DC.	36	2*n* = 28m + 8sm	98.30	0.33	0.13	2A	2G, S3C
*A. moorcroftiana* Wall. ex DC.	36	2*n* = 28m + 8sm	101.14	0.31	0.14	2A	2H, S3D
*A. moorcroftiana* Wall. ex DC.	36						2I
*A. moorcroftiana* Wall. ex DC.	36						2J
*A. phaeolepis* Krasch	36	2*n* = 28m + 8sm	132.9	0.20	0.15	2B	2K, S3E
*A. phaeolepis* Krasch	18	2*n* = 12m + 6sm	66.43	0.30	0.13	2A	2L, S3F
*A. princeps* Pamp.	32	2*n* = 28m + 4st	80.26	0.30	0.16	2A	2M, S3G
*A. qinlingensis* Ling & Y.R. Ling	18	2*n* = 14m + 4st	52.50	0.34	0.17	2A	2N, S3H
*A. qinlingensis* Ling & Y.R. Ling	18	2*n* = 12m + 6sm	67.54	0.35	0.10	2A	2O, S4A
*A. qinlingensis* Ling & Y.R. Ling	18						2P
*A. sacrorum* Ledeb.	18	2*n* = 14m + 4sm	68.04	0.26	0.08	2A	3A, S4B
*A. tainingensis* Hand.-Mazz.	36						3B
*A. tainingensis* Hand.-Mazz.	36	2*n* = 28m + 4sm + 4st	84.77	0.30	0.13	2A	3C, S4C
*A. tainingensis* Hand.-Mazz.	36	2*n* = 28m + 8st	94.25	0.32	0.14	2A	3D, S4D
*A. tainingensis* Hand.-Mazz.	36						3E
*A. verbenacea* (Komar.) Kitag.	16	2*n* = 14m + 2sm	59.34	0.28	0.20	2A	3F, S4E
*A. verbenacea* (Komar.) Kitag.	16	2*n* = 14m + 2st	65.88	0.24	0.18	2A	3G, S4F
*A. verbenacea* (Komar.) Kitag.	16	2*n* = 14m + 2st	69.89	0.26	0.14	2A	3H, S4G
*A. verlotiorum* Lamotte	50	2*n* = 40m + 10st	133.18	0.25	0.19	2A	3I, S4H
*A. verlotiorum* Lamotte	50						3J
*A. verlotiorum* Lamotte	50						3K
*A. verlotiorum* Lamotte	50						3L
*A. vulgaris* L.	16	2*n* = 14m + 2st	71.00	0.23	0.12	2A	3M, S5A
*A*. *vulgaris* var. *xizangensis* Ling & Y.R. Ling	36	2*n* = 28m + 4sm + 4st	97.07	0.30	0.14	2A	3N, S5B
*A. waltonii* var. *yushuensis* Y. R. Ling	36	2*n* = 28m + 8sm	110.62	0.29	0.10	2A	3O, S5C
*A. waltonii* var. *yushuensis* Y. R. Ling	36						3P
*A. waltonii* var. *yushuensis* Y. R. Ling	36						4A,
*A. wellbyi* Hemsl. & Pears. ex Deasy	18	2*n* = 14m + 2sm + 2st	66.74	0.28	0.11	2A	4B, S5D
*A. zayuensis* Ling & Y. R. Ling	18	2*n* = 12m + 6sm	64.25	0.29	0.16	2A	4C, S5E
*A. younghusbandii* J. R. Drumm. ex Pamp.	18	2*n* = 14m + 4sm	61.14	0.29	0.14	2A	4D, S5F
*A. youngii* Y. R. Ling	18						4E
*A. youngii* Y. R. Ling	18	2*n* = 12m + 6sm	68.68	0.32	0.13	2A	4F, S5G
*A. youngii* Y. R. Ling	18						4G,
*A. yunnanensis* J.F. Jeffrey ex Diels	32	2*n* = 24m + 4sm + 4st	73.19	0.30	0.19	2B	4H, S5H

## 4. Discussion

In this study, the chromosome numbers of 56 populations in 25 species and four varieties of *Artemisia* from China were examined. Most species studied here are diploid (2*n* = 18) or tetraploid (2*n* = 36) based on *x* = 9, further confirming *x* = 9 as the most common chromosome base number in *Artemisia* [15,16,17,21]. Another basic chromosome number, *x* = 8, is rarely reported [15]. Only four species are based on *x* = 8, with *A. verbenacea* (Komar.) Kitag. and *A. vulgaris* L. being diploid (2*n* = 16), and *A. princeps* Pamp. and *A. yunnanensis* J.F. Jeffrey ex Diels being tetraploid (2*n* = 32). All four of these species belong to subg. *Artemisia*.

The chromosome numbers of some species have been reported in previous studies. Three cytotypes have been consecutively reported for *A. lactiflora* from China, including 2*n* = 18 [49], 36, and 54 [23]. Only chromosome numbers of 2*n* = 18 and 36 were found here. *Artemisia igniaria* is mainly distributed in northern China. Wang et al. revealed that one population from Inner Mongolia in China was aneuploid, with 2*n* = 34, and the karyotype was 2*n* = 28m + 2sm + 4st (4sat) [50]. This chromosome number was further confirmed in the Hebei population but with a different karyotype (2*n* = 30m + 4sm). Kaul and Bakshi reported 2*n* = 16 for a population of *A. incisa* from Kashimir [51]. However, we found that the population from Xizang was tetraploid (2*n* = 36), representing a new cytotype for this species. *Artemisia gmelinii* and *A. sacrorum* were morphologically similar. Populations of these species from China were cytologically studied here for the first time, and both were found to be diploid, with 2*n* = 18 and 16, respectively. The Russian populations of *A*. *gmelinii* were previously reported to be 2*n* = 36 [19] and 54 [18], and the population from South Korea was found to be 2*n* = 54 [25]. For *A*. *sacrorum*, only tetraploid or hexaploid populations were reported from Russia, South Korea, and Italy. *Artemisia minor* and *A. moorcroftiana* are endemic to QTP and have been reported to be diploid (2*n* = 18) [51,52,53,54]. However, we found that four populations of *A. moorcroftiana* from Xizang in China were all tetraploid (2*n* = 36), representing a new cytotype for this species. *Artemisia phaeolepis* was reported to have two cytotypes (2*n* = 18, 36) [55], and *A. anomala* was diploid (2*n* = 18) [56]; this result was confirmed in this study. Populations of *A. princeps* from Japan have been documented to be aneuploid, with 2*n* = 34 [23,27,49,57,58]. We found one population from Sichuan in China to be tetraploid, with 2*n* = 4*x* = 32, representing a new cytotype for this species. *Artemisia vulgaris* is widely distributed in the Northern Hemisphere. Populations from Russia are diploid, with 2*n* = 16 [18,54,59,60,61,62,63] or 18 [18], and populations from Britain, France, Germany, India, and Iran have been shown to be 2*n* = 16 [29,59,64,65,66,67]. The chromosome number of this species from China is reported here for the first time and was also found to be 2*n* = 16.

Previous studies have indicated that *A. verlotiorum* had different cytotypes. Populations from Italia were reported to be 2*n* = 48 and 54 [31,64]; populations from Britain were 2*n* = 48, 50, and 52 [32]; and those from Russia and Germany were 2*n* = 54 [33,68]. Due to the remarkable discrepancy, some chromosome numbers reported by previous authors (including 2*n* = 87, 88, 89) are quite doubtful and need to be verified [69]. Four populations of *A*. *verlotiorum* (2*n* = 50) from China were found to be aneuploid. A total of 12 plants were examined and confirmed this result. It is noteworthy that misidentifications in *A. verlotiorum* are very common. The vouchers of previous studies should be carefully checked to verify the results.

The chromosome number is stable within some species and has taxonomic value. *Artemisia taibaishanensis* Y.R. Ling & Ling was originally recognized as an independent species. Based on observations of herbarium specimens (including type material) and living plants in the wild, Guo et al. demonstrated that it is conspecific with *A. qinlingensis*, and therefore placed *A. taibaishanensis* in the synonymy of *A. qinlingensis* [70]. We cytologically examined three populations of *A*. *qinlingensis*, with one population from the type locality, Luanchuan, in Henan, and one population from the type locality of *A. taibaishanensis*, Meixian in Shaanxi. The results indicated that the chromosome numbers were stable within *A. qinlingensis*, and three populations were diploid, with a chromosome number of 2*n* = 18. Thus, the cytological data were consistent with the taxonomic treatment by Guo et al. [70]. *Artemisia youngii* is morphologically similar to *A*. *imponens*, *A*. *moorcroftiana*, and *A*. *tainingensis*. These species also overlap in geographical distribution and are difficult to distinguish from each other. Based on examination of specimens and observation of living plants, we found that *A. youngii* differed from *A*. *imponens*, *A*. *moorcroftiana*, and *A*. *tainingensis* in having linear-lanceolate leaf lobules (vs. lanceolate). This result was further confirmed in this research. *A*. *youngii* was found to be diploid, with 2*n* = 18, while the other three species were found to be tetraploid, with 2*n* = 36. Until now, the cytological data of Chinese *Artemisia* is still not adequate; further studies are still needed to fully understand the variation pattern of chromosomal data and evaluate their taxonomic and phylogenetic values.

Of the 25 species and four varieties cytologically studied, 11 species and 3 varieties are tetraploid or have tetraploid cytotypes. These include: *A. baimaensis* Y.R. Ling & Z.C. Chuo, (2*n* = 36); *A. campbellii*, (2*n* = 36); *A. incisa*, (2*n* = 36); *A. imponens* Pamp., (2*n* = 36); *A. jilongensis* Y.R. Ling & Humphries, (2*n* = 36); *A. lactiflora* var. *incisa* (Pamp.) Y.R. Ling, (2*n* = 36); *A. moorcroftiana*, (2*n* = 36); *A. phaeolepis*, (2*n* = 36); *A. princeps*, (2*n* = 32); *A. tainingensis*, (2*n* = 36); *A. vulgaris* var. *xizangensis* Ling & Y.R. Ling, (2*n* = 36); *A. waltonii* var. *yushuensis* Y.R. Ling, (2*n* = 36); and *A. yunnanensis* J.F. Jeffrey ex Diels, (2*n* = 32). Among them, *A. baimaensis*, *A. campbellii*, *A. incisa*, *A. imponens*, *A. jilongensis*, *A. moorcroftiana*, *A. tainingensis*, *A. vulgaris* var. *xizangensis*, *A. waltonii* var. *yushuensis*, and *A. yunnanensis* are endemic to the QTP [3]. It is worth mentioning that two cytotypes were found in *A. phaeolepis* in China. The tetraploid population was found in the QTP, whereas the diploid population occurs outside this region. This surprisingly high rate of polyploid strongly suggests that polyploidy may have played an important role in the evolutionary speciation of *Artemisia* in this region [34,43]. Similar results have also been reported for the genera *Buddleja*, *Leontopodium* R. Br. ex Cass., and *Silene* L. [44,71,72]. Several groups, including *Crementhodium*, *Delphinium*, and *Ligularia*, however, show a low rate of polyploidy in the QTP [43,45,47]. In these groups, polyploidy may have played a minor role in the evolutionary diversification in the QTP. The chromosomal data from the QTP are still inadequate, and further cytological investigations are still needed to evaluate the role of polyploidy in the speciation in the QTP.

The karyotypic features of the *Artemisia* species studied here are similar. Most chromosomes are median-centromeric, and a few chromosomes are submedian-centromeric or subterminal-centromeric. In diploids, the most common karyotype formula is 2*n* = 14m + 4sm, and in tetraploids, the most common is 2*n* = 28m + 8sm. No essential difference was found between the species, which are morphologically similar. All the investigated species display relatively primitive 2A asymmetry, except for *A. yunnanensis*, which showed 2B asymmetry. In this study, only one plant in the Lushi population of *A. verlotiorum* was found to have three satellite chromosomes (Figure 3J). However, the satellite chromosomes are not stable even within the same population. The chromosomes of diploid taxa are usually larger than those of tetraploid taxa. The average length range of chromosomes in diploids is 2.9–4.7 μm, and that of chromosomes in tetraploids is 2.2–3.7 μm. However, in the diploid and tetraploid populations of *A. phaelopis*, there is no essential difference in the length of the chromosomes. In terms of the high proportion of m and sm chromosomes, the karyotypes of *Artemisia* species are symmetrical, with low values of A1 and A2. Due to the uniform karyotype of this genus, it is still difficult to infer the taxonomic and phylogenetic relationship between closely related taxa solely based on karyotypic evidence.

## Data Availability

Data are contained within the article.

## Supplementary Materials

The following supporting information can be downloaded at: https://www.mdpi.com/article/10.3390/plants14081253/s1. Figure S1. Karyotypes of nine populations representing six species and one variety in *Artemisia* from China. A. *A. anomala*, 2*n* = 18; B. *A. anomala*, 2*n* = 18; C. *A. baimaensis*, 2*n* = 36; D. *A. campbellii* 2*n* = 36; E. *A. divaricata*, 2*n* = 18; F. *A. divaricata*, 2*n* = 18; G. *A. fulgens* var. *meiguensis*, 2*n* = 18; H. *A. incisa*, 2*n* = 36; I. *A*. *igniaria*, 2*n* = 34. All same scale. Figure S2. Karyotypes of six populations representing two species and one variety in *Artemisia* from China. A. *A. imponens*, 2*n* = 36; B. *A. imponens*, 2*n* = 36; C. *A. imponens*, 2*n* = 36; D. *A. lactiflora*, 2*n* = 18; E. *A. lactiflora*, 2*n* = 18; F. *A. lactiflora* var. *incisa*, 2*n* = 36. All same scale. Figure S3. Karyotypes of seven populations representing five species and one variety in *Artemisia* from China. A. *A. lactiflora* var. *incisa*, 2*n* = 36; B. *A. minor*, 2*n* = 18; C. *A. moorcroftiana*, 2*n* = 36; D. *A. moorcroftiana*, 2*n* = 36; E. *A. phaeolepis*, 2*n* = 36; F. *A. phaeolepis*, 2*n* = 18; G. *A. princeps*, 2*n* = 32; H. *A. qinlingensis*, 2*n* = 18. All same scale. Figure S4. Karyotypes of eight populations representing five species in *Artemisia* from China. A. *A. qinlingensis*, 2*n* = 18; B. *A. sacrorum*, 2*n* = 18; C. *A. tainingensis*, 2*n* = 36; D. *A. tainingensis*, 2*n* = 36; E. *A. verbenacea*, 2*n* = 16; F. *A. verbenacea*, 2*n* = 16; G. *A. verbenacea*, 2*n* = 16; H. *A. verlotiorum*, 2*n* = 50. All same scale. Figure S5. Karyotypes of eight populations representing five species in *Artemisia* from China. A. *A. vulgaris*, 2*n* = 16; B. *A. vulgaris* var. *xizangensis*, 2*n* = 36; C. *A. waltonii* var. *yushuensis,* 2*n* = 36; D. *A. wellbyi*, 2*n* = 18; E. *A. zayuensis*, 2*n* = 18; F. *A. younghusbandii*, 2*n* = 18; G. *A. youngii*, 2*n* = 18; H. *A. yunnanensis*, 2*n* = 32. All same scale. Table S1. Source of material in each taxon investigated within *Artemisia* from China.

## Abbreviations

QTP	Qinghai–Tibetan Plateau
m	median-centromeric chromosome
sm	submedian-centromeric chromosome
st	subterminal-centromeric chromosome
