# Supplementary material for: Cytological Studies of 25 Species and Four Varieties of Artemisia (Asteraceae) from China, Toward a Better Understanding of the Variation Patterns of Chromosomes in the Genus
Source: Plants (Basel). 2025 Apr 20;14(8):1253. doi: 10.3390/plants14081253 (PMC12030662; doi:10.3390/plants14081253)
Supplement: Supplementary file 1 [file plants-14-01253-s001.zip › plants-3573034-supplementary.pdf]

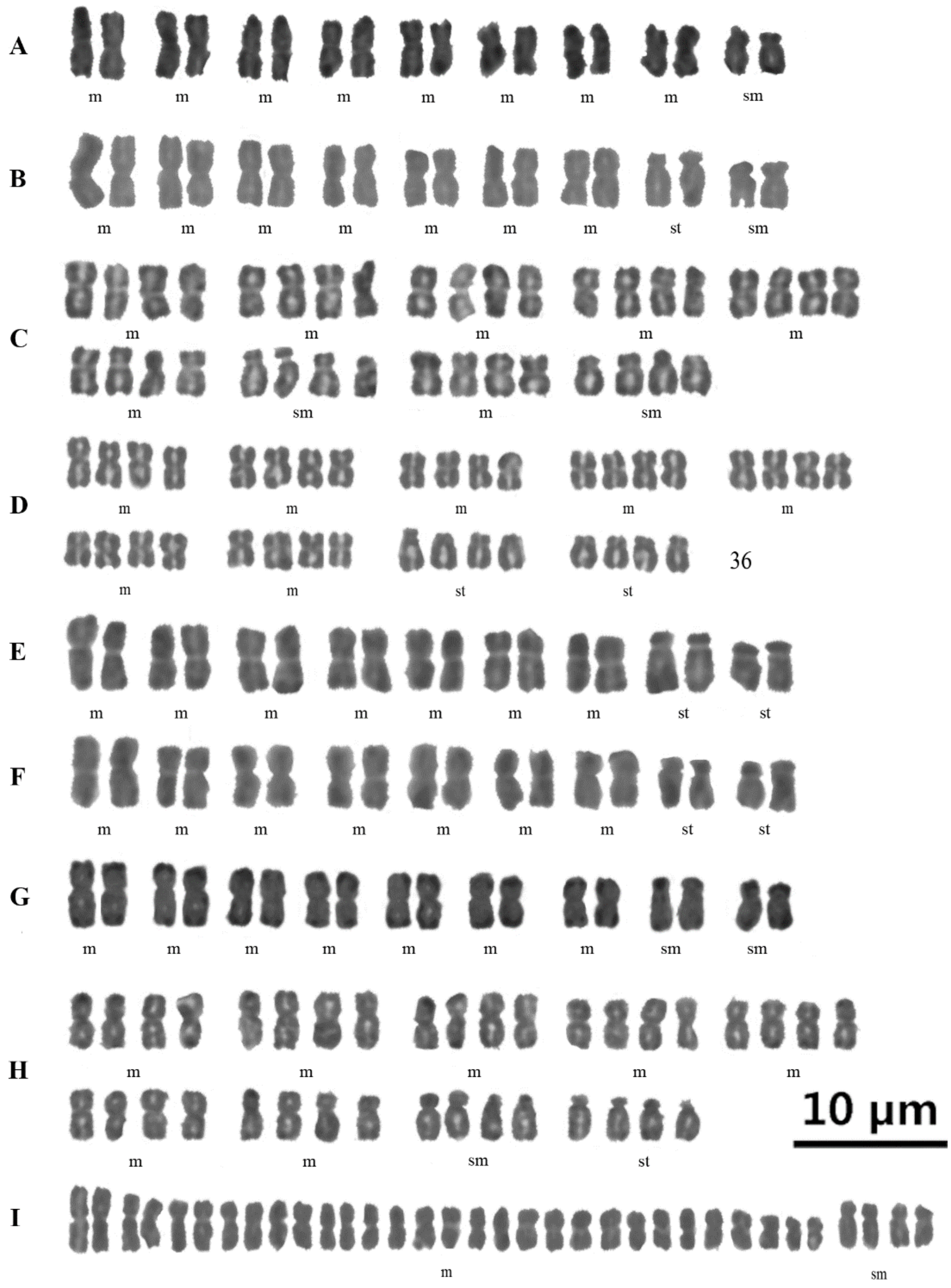

Figure S1. Karyotypes of nine populations representing six species and one variety in *Artemisia* from China. A. *A. anomala*, 2*n* = 18; B. *A. anomala*, 2*n* = 18; C. *A. baimaensis*, 2*n* = 36; D. *A. campbellii* 2*n* = 36; E. *A. divaricata*, 2*n* = 18; F. *A. divaricata*, 2*n* = 18; G. *A. fulgens* var. *meiguensis*, 2*n* = 18; H. *A. incisa*, 2*n* = 36; I. *A. igniaria*, 2*n* = 34. All same scale.

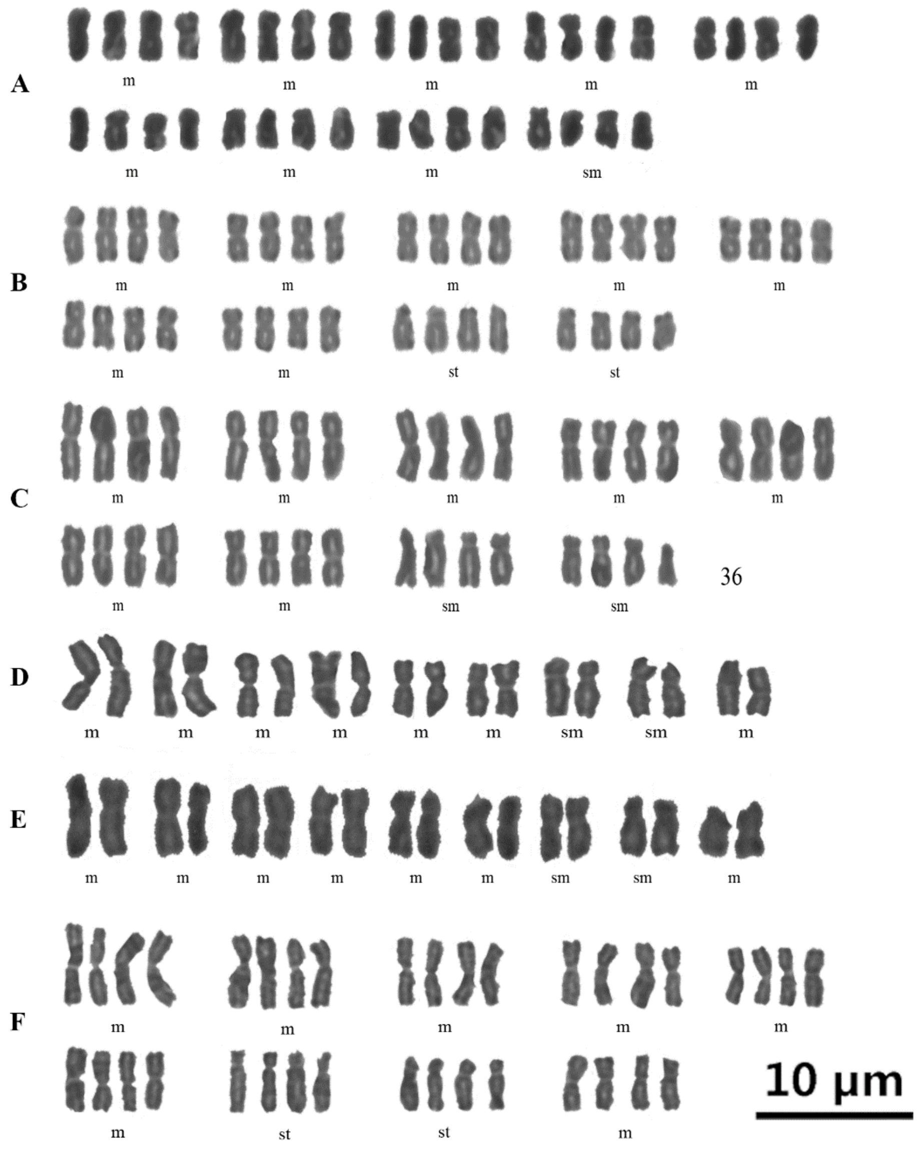

Figure S2. Karyotypes of six populations representing two species and one variety in *Artemisia* from China. A. *A. imponens*, 2*n* = 36; B. *A. imponens*, 2*n* = 36; C. *A. imponens*, 2*n* = 36; D. *A. lactiflora*, 2*n* = 18; E. *A. lactiflora*, 2*n* = 18; F. *A. lactiflora* var. *incisa*, 2*n* = 36. All same scale.

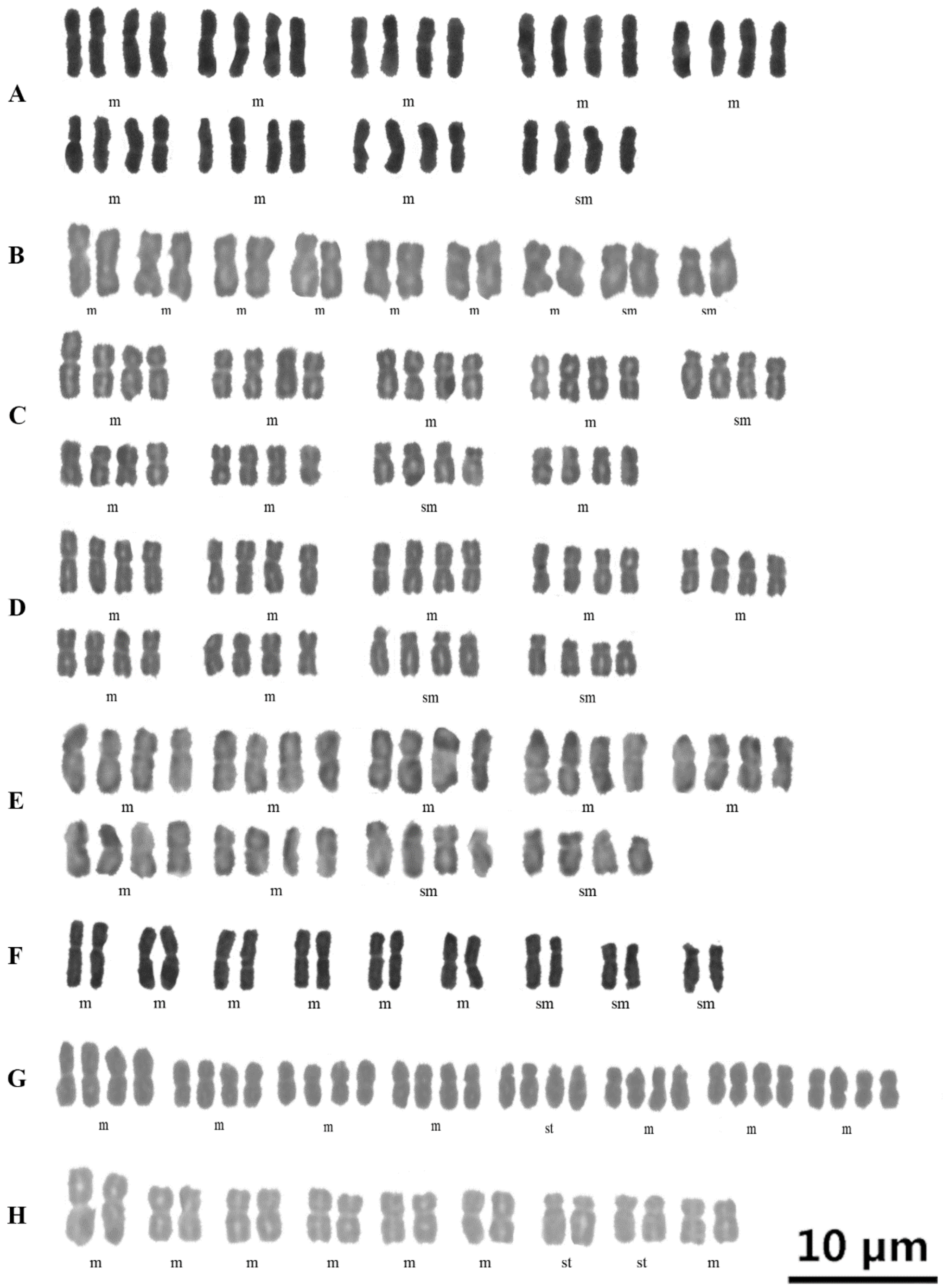

Figure S3. Karyotypes of seven populations representing five species and one variety in *Artemisia* from China. A. *A. lactiflora* var. *incisa,* 2*n* = 36; B. *A. minor,* 2*n* = 18; C. *A. moorcroftiana,* 2*n* = 36; D. *A. moorcroftiana,* 2*n* = 36; E. *A. phaeolepis,* 2*n* = 36; F. *A. phaeolepis,* 2*n* = 18; G. *A. princeps,* 2*n* = 32; H. *A. qinlingensis,* 2*n* = 18. All same scale.

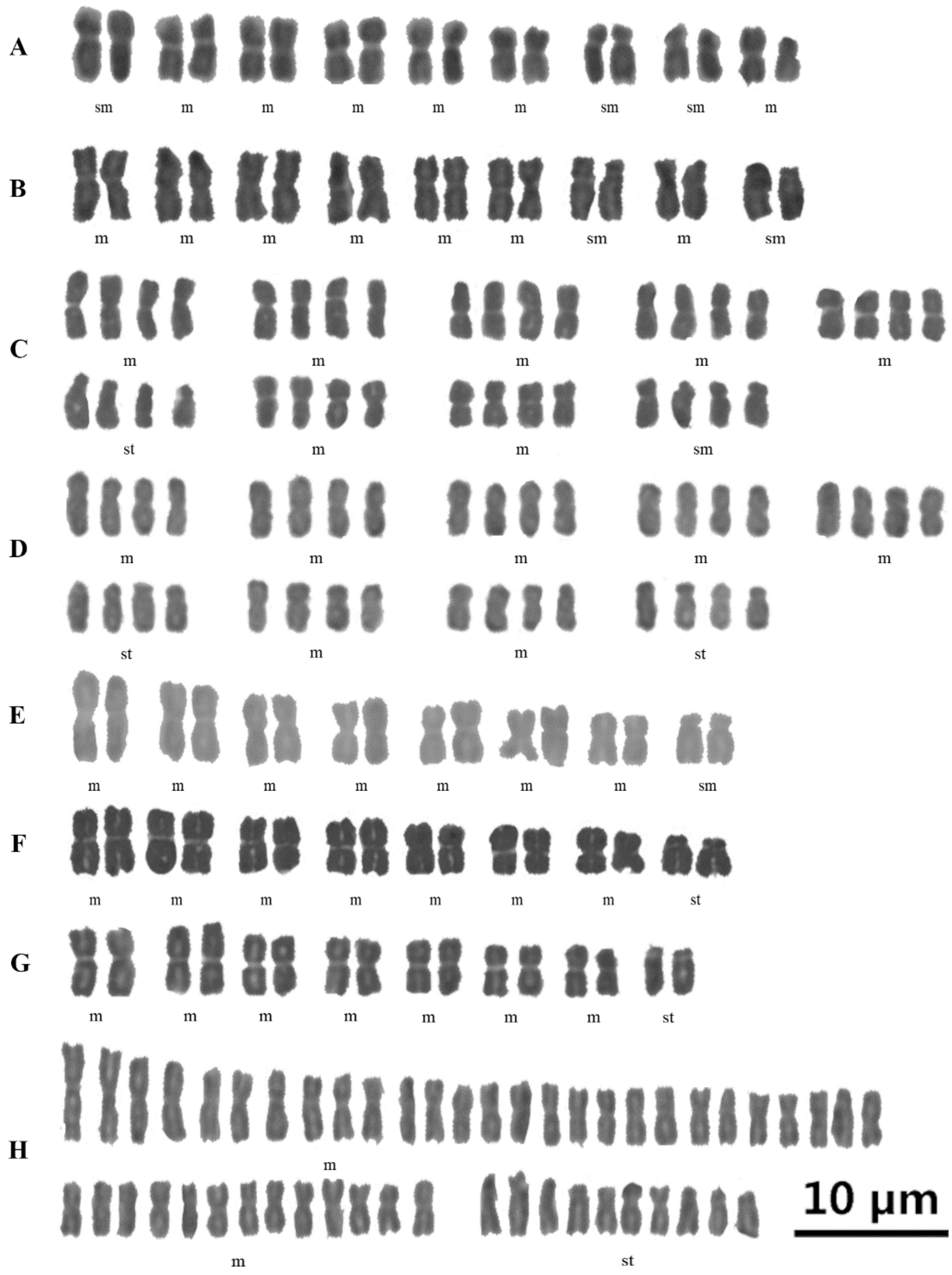

Figure S4. Karyotypes of eight populations representing five species in *Artemisia* from China. A. *A. qinlingensis*, 2*n* = 18; B. *A. sacrorum*, 2*n* = 18; C. *A. tainingensis*, 2*n* = 36; D. *A. tainingensis*, 2*n* = 36; E. *A. verbenacea*, 2*n* = 16; F. *A. verbenacea*, 2*n* = 16; G. *A. verbenacea*, 2*n* = 16; H. *A. verlotiorum*, 2*n* = 50. All same scale.

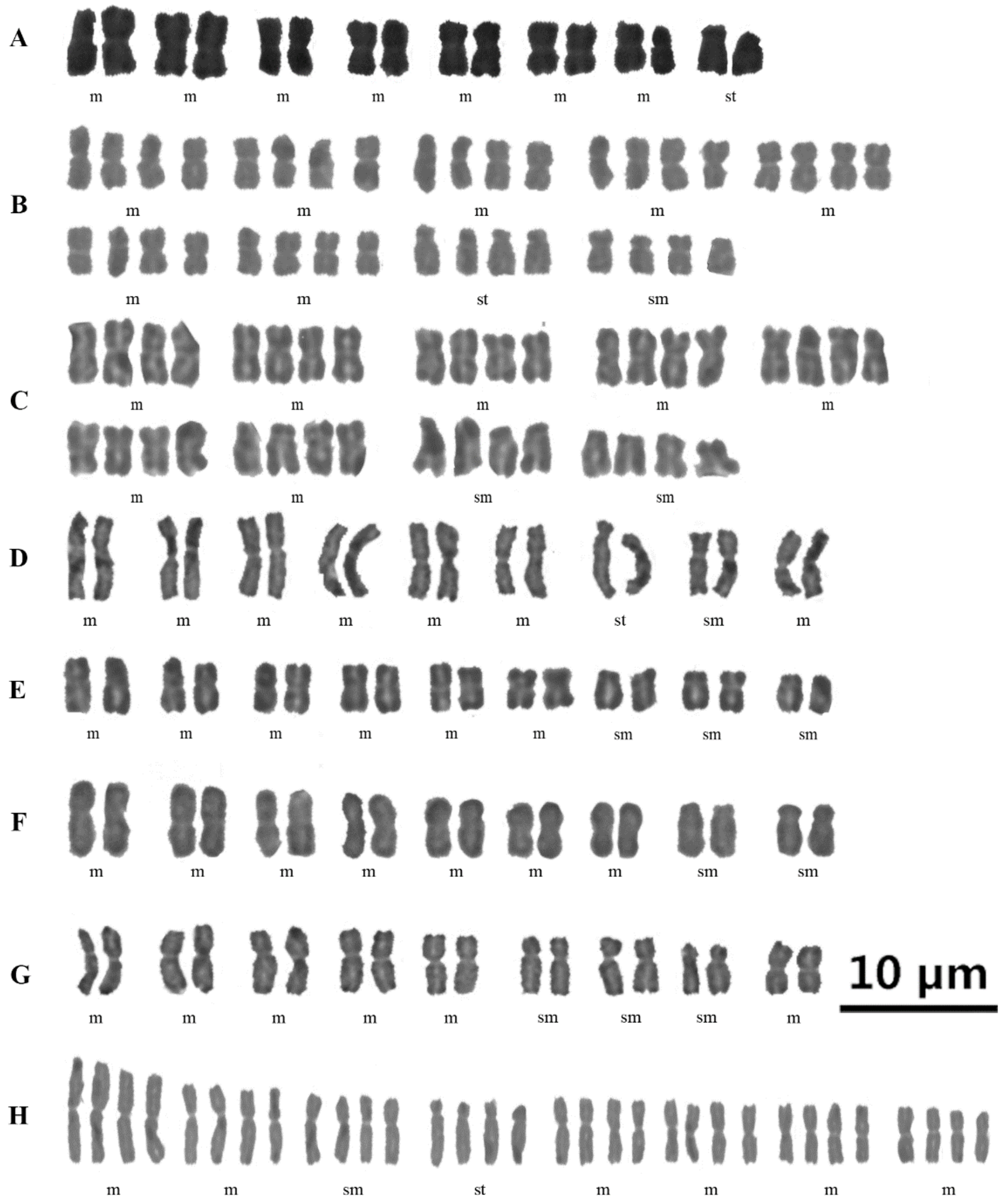

Figure S5. Karyotypes of eight populations representing five species in *Artemisia* from China. A. *A. vulgaris*, 2*n* = 16; B. *A. vulgaris* var. *xizangensis*, 2*n* = 36; C. *A. waltonii* var. *yushuensis,* 2*n* = 36; D. *A. wellbyi*, 2*n* = 18; E. *A. zayuensis*, 2*n* = 18; F. *A. younghusbandii*, 2*n* = 18; G. *A. youngii*, 2*n* = 18; H. *A. yunnanensis*, 2*n* = 32. All same scale.

Table S1. Source of material in each taxon investigated within *Artemisia* from China.

Taxon	Locality	Longitude	Latitude	Voucher
A. anomala S. Moore	Guangdong, Yingde	113°32'55.42'' E	24°16'43.59'' N	Y.P. Zeng 279
A. anomala S. Moore	Hunan, Xinning	110°37'12.58'' E	26°30'34.41'' N	Y.P. Zeng 80
A. anomala S. Moore	Zhejiang, Hangzhou	120°6'6.822'' E	30°14'13.09'' N	L. Wang et al. 4226
A. baimaensis Y.R. Ling & Z.C. Chuo	Qinghai, Baima	100°52'21.64'' E	32°43'18.93'' N	L. Wang et al. 3591
A. campbellii Hook. f. & Thoms.	Xizang, Lhünzê	92°53'11.12'' E	28°32'41.80'' N	L. Wang et al. 3019
A. divaricata (Pamp.) Pamp.	Sichuan, Barkam	102°20'10.89'' E	31°51'56.16'' N	L. Wang et al. 3664
A. divaricata (Pamp.) Pamp.	Sichuan, Jinchuan	102°04'12.68'' E	31°26'05.83'' N	L. Wang et al. 3668
A. divaricata (Pamp.) Pamp.	Sichuan, Zamtang	100°55'01.56'' E	32°42'15.18'' N	L. Wang et al. 3580
A. fulgens var. *meiguensis* (Y.R. Ling) X.Q. Guo, L. Wang & Q.E. Yang	Sichuan, Ebian	103°03'41.46'' E	28°42'05.74'' N	L. Wang & X.Q. Guo 2216
A. gmelinii Web. ex Stechm.	Qinghai, Madoi	98°28'44.59'' E	34°52'44.20'' N	L. Wang et al. 4076
A. incisa Pamp.	Xizang, Dinggyê	87°27'21.90'' E	27°54'41.79'' N	L. Wang et al. 2853
A. igniaria Maxim.	Hebei, Xingtai	113°58'26.79'' E	37°24'17.00'' N	L. Wang et al. 4194
A. imponens Pamp.	Sichuan, Hongyuan	102°42'50.96'' E	32°54'52.86'' N	L. Wang et al. 3652
A. imponens Pamp.	Sichuan, Songpan	102°23'30.00'' E	32°55'20.27'' N	L. Wang et al. 3606
A. imponens Pamp.	Sichuan, Xiangcheng	99°44'31.40'' E	29°00'03.57'' N	L. Wang et al. 3693
A. jilongensis Y. R. Ling & Humphries	Xizang, Gyirong	85°13'01.94'' E	28°31'11.78'' N	L. Wang et al. 2721
A. lactiflora Wall. ex DC.	Sichuan, Baoxing	102°32'44.05'' E	30°27'15.13'' N	J.P. Luo 211
A. lactiflora Wall. ex DC.	Sichuan, Dayi	103°09'45.25'' E	30°40'40.26'' N	J.P. Luo 536
A. lactiflora Wall. ex DC.	Hubei, Changyang	111°01'11.95'' E	30°29'47.11'' N	L. Wang et al. 4221
A. lactiflora var. *taibaishanensis* X.D. Cui	Hubei, Shennongjia	110°20'22.75'' E	31°33'36.49'' N	J.P. Luo 176
A. lactiflora var. *taibaishanensis* X.D. Cui	Sichuan, Tianquan	102‘18’42.47'' E	29‘51’33.06'' N	J.P. Luo 418
A. minor Jacq. ex Bess.	Qinghai, Madoi	98°28'44.59'' E	34°52'44.20'' N	L. Wang et al. 4078

A. moorcroftiana Wall. ex DC.	Xizang, Baxoi	97°34'17.90'' E	29°55'52.58'' N	L. Wang & X.Q. Guo 3380
A. moorcroftiana Wall. ex DC.	Xizang, Bomi	95°45'59.21'' E	29°51'17.77'' N	W. Q. Fei 63
A. moorcroftiana Wall. ex DC.	Xizang, Comai	91°40'35.52'' E	28°36'35.19'' N	L. Wang et al. 2490
A. moorcroftiana Wall. ex DC.	Xizang, Zayü	97°08'05.73'' E	29°19'21.62'' N	L. Wang & X.Q. Guo 2422
A. phaeolepis Krasch	Qinghai, Maqén	100°35'31.16'' E	34°38'36.30'' N	L. Wang et al. 4095
A. phaeolepis Krasch	Xinjiang, Huocheng	81°20'24.78'' E	44°27'27.59'' N	C. Ren & L. Wang 854
A. princeps Pamp.	Sichuan, Luding	102°14'37.63'' E	29°49'11.26'' N	L. Wang et al. 3681
A. qinlingensis Ling & Y.R. Ling	Henan, Luanchuan	111°38'45.54'' E	33°43'12.11'' N	L. Wang et al. 4182
A. qinlingensis Ling & Y.R. Ling	Hubei, Shennongjia	110°20'22.75'' E	31°33'36.49'' N	J.P. Luo 171
A. qinlingensis Ling & Y.R. Ling	Shaanxi, Meixian	107°44'46.39'' E	33°59'15.96'' N	L. Wang et al. 4168
A. sacrorum Ledeb.	Xinjiang, Ürümqi	81°20'24.78'' E	44°27'27.59'' N	C. Ren & L. Wang 726
A. tainingensis Hand.-Mazz.	Qinghai, Henan	102°05'55.08'' E	34°25'38.90'' N	L. Wang et al. 4101
A. tainingensis Hand.-Mazz.	Qinghai, Madoi	98°28'44.59'' E	34°52'44.20'' N	L. Wang et al. 4077
A. tainingensis Hand.-Mazz.	Sichuan, Aba	101°01'25.10'' E	30°45'02.95'' N	L. Wang et al. 3595
A. tainingensis Hand.-Mazz.	Xizang, Jomda	97°50'12.05'' E	31°23'28.62'' N	L. Wang et al. 3423
A. verbenacea (Komar.) Kitag.	Sichuan, Dawu	101°07'57.01'' E	30°59'46.63'' N	L. Wang & X.Q. Guo 2340
A. verbenacea (Komar.) Kitag.	Sichuan, Jiuzhaigou	103°48'04.42'' E	33°18'41.49'' N	L. Wang et al. 3635
A. verbenacea (Komar.) Kitag.	Xizang, Qamdo	97°19'58.74'' E	31°30'24.37'' N	L. Wang et al. 3412
A. verlotiorum Lamotte	Henan, Lushi	110°48'57.35'' E	33°45'00.64'' N	J.P. Luo 165
A. verlotiorum Lamotte	Shaanxi, Meixian	107°40'05.16'' E	34°03'00.72'' N	L. Wang et al. 4179
A. verlotiorum Lamotte	Hubei, Shennongjia	110°20'22.75'' E	31°33'36.49'' N	J.P. Luo 172
A. verlotiorum Lamotte	Sichuan, Songpan	103°45'58.33'' E	32°12'36.43'' N	L. Wang et al. 3648
A. vulgaris L.	Xinjiang, Ürümqi	87°10'13.16'' E	43°16'03.46'' N	C. Ren & L. Wang 701
A. vulgaris var. *xizangensis* Ling & Y.R. Ling	Xizang, Bomi	96°16'40.37'' E	29°40'38.66'' N	L. Wang et al. 3322
A. waltonii var. *yushuensis* Y. R. Ling	Qinghai, Chindu	97°17'25.54'' E	33°00'03.33'' N	L. Wang et al. 3522
A. waltonii var. *yushuensis* Y. R. Ling	Qinghai, Qumarlêb	95°52'42.24'' E	34°08'24.21'' N	L. Wang et al. 3528

A. waltonii var. *yushuensis* Y. R. Ling	Xizang, Gongjo	98°15'30.28'' E	30°53'54.55'' N	L. Wang & X.Q. Guo 2386
A. wellbyi Hemsl. & Pears. ex Deasy	Xizang, Dinggyê	87°41'53.58'' E	28°07'41.36'' N	L. Wang et al. 2827
A. zayuensis Ling & Y. R. Ling	Xizang, Lhünzê	93°07'08.93'' E	28°30'14.12'' N	L. Wang et al. 3051
A. younghusbandii J. R. Drumm. ex Pamp.	Xizang, Kangmar	89°23'51.46'' E	28°16'48.56'' N	L. Wang et al. 2509
A. youngii Y. R. Ling	Qinghai, Nangqên	96°22'45.18'' E	32°00'21.70'' N	L. Wang et al. 3510
A. youngii Y. R. Ling	Xizang, Baxoi	97°28'35.40'' E	29°56'38.29'' N	L. Wang et al. 3381
A. youngii Y. R. Ling	Xizang, Riwoqê	96°30'57.65'' E	31°22'23.11'' N	L. Wang et al. 3490
A. yunnanensis J.F. Jeffrey ex Diels	Sichuan, Danba	101°57'07.70'' E	30°47'35.51'' N	L. Wang et al. 3680

Notes: all the vouchers were deposited in Herbarium of South China Botanical Garden, Chinese Academy of Sciences (IBSC).
